# Population-level prevalence, effect on quality of life, and treatment behavior for erectile dysfunction and premature ejaculation in Poland

**DOI:** 10.1038/s41598-023-39968-9

**Published:** 2023-08-14

**Authors:** Mikolaj Przydacz, Marcin Chlosta, Pawel Rajwa, Piotr Chlosta

**Affiliations:** 1 Department of Urology, Jagiellonian University Medical College, ul. Macieja Jakubowskiego 2, 30-688 Krakow, Poland; 2 Department of Urology, Medical University of Silesia, Zabrze, Poland; 3 Department of Urology, Comprehensive Cancer Center, Medical University of Vienna, Vienna, Austria

**Keywords:** Urology, Public health

## Abstract

The prevalence of erectile dysfunction (ED) and premature ejaculation (PE) has been investigated in many population-based studies in different regions of the world. However, reliable data are lacking for Eastern Europe. Therefore, the aim of this study was to analyze the prevalence, effect on quality of life, and treatment-related behaviors for ED and PE in a population-representative sample of Polish men. We used an Internet interview format and rigorously adapted, widely accepted instruments for ED and PE evaluation. The study included 3001 men, representative for age and place of residence and adequate proportions of respondents from urban and rural areas. The prevalence of ED was 30.1–61.1%, and the prevalence of PE was 19.3–38.1%; there were no differences between urban and rural areas. Whereas the prevalence of ED increased with age, the prevalence of PE did not increase. More than 50% of respondents with ED and more than 60% of respondents with PE had concerns about their quality of life. However, less than one fourth of participants with ED and PE were seeking treatment, most of whom received treatment. The results of our nationwide analysis, reflecting the entire Polish population of men, are consistent with other epidemiologic studies of ED and PE and may support educational campaigns and health improvement programs in Poland.

## Introduction

Erectile dysfunction (ED) and premature ejaculation (PE) represent significant concerns in the sexual life of men. Both conditions lead to multiple issues in personal and social functioning, impairment of physical, mental, and emotional health, and finally to decreased overall well-being and quality of life^1^.

A substantial group of large epidemiological studies have been focused on evaluating ED and PE at the population level in many parts of the world. Perhaps interestingly, these studies yielded disparate results, with the overall ED prevalence varying from 3 to 76.5%^2^, and the overall PE prevalence being from 2 to 60%^3^. Such wide disparities are often due to differences in the selected study population, e.g., hospital- versus community- versus population-based, age, survey methodology, participant population size, data collection method, definition of disorder or symptoms, and culture or ethnicity.

Despite the many existing epidemiological studies, reliable data for ED and PE are still lacking for Central and Eastern Europe. Even in large-scale and population-representative European studies conducted to ascertain the prevalence of ED or PE, countries from Central and Eastern Europe have not been included^2,4^. Although there are some population-level analyses on ED from Central (e.g., Austria^5^) and Eastern (e.g., Russia^6^) Europe, these studies have been performed in single cities (e.g., the Austrian study was limited to the Vienna area^5^) or burdened with a significant selection bias in terms of sample representativeness (e.g., the Russian study included patients presenting to hospitals and outpatient departments for any illness^6^). In sum, no large population-representative study in any country of this region has been conducted to reliably evaluate the prevalence of ED and PE. Indeed, experts stipulate that, for high-quality research, investigators should use generally accepted and professionally adapted instruments, ideally in surveying representative pools^7^. Population estimates of ED and PE are needed to increase general awareness, promote physical and mental health, and start appropriate educational programs. These data also tempt multidisciplinary frameworks for national health improvement programs and service deliveries instituted with appropriate allocation of resources by governments and healthcare systems.

Poland is the largest country in Central Europe^8,9^; by land area, Poland is the third largest in Eastern Europe, after Russia and Ukraine, and the farthest east of the European Union countries^10^. Until now, no reliable and population representative study on ED and PE has been conducted in Poland. Poland and other Central and Eastern European countries are demographically unique, having homogeneous or even supra-ethnic uniformity; particularly for Poland, ≥ 99% of residents are Caucasian and ≥ 90% of residents are of Polish identity^11^. Such unique population features need to be considered when discussing population-based studies for any symptoms or disorders. Further, healthcare-related behavior may vary significantly between countries and regions because of local cultural norms that may inhibit individuals from admitting or discussing their health issues, especially when it comes to sensitive topics like sexual life^12^. Indeed, Central-Eastern Europe is often considered a distinct cultural entity, and Slavic people are culturally different from other European people^13^. Additionally, a relatively high number of people in Poland live in rural regions. Most data from elsewhere on prevalence, burden, and treatment-related behaviors for ED and PE may not be fully transferable to Poland because the epidemiological data originate from industrialized areas, including data publicized as being from low- or middle-income countries, with no comparisons between urban and rural areas. An analysis of treatment behaviors is particularly important for ED because medical attention for this condition may uncover other serious and asymptomatic morbidities early in the course of disease (e.g., diabetes, hypertension, coronary artery disease, depression)^1^. Considering all these factors, our understanding of the prevalence and true burden of ED and PE in Poland is limited. Fortunately, the importance of population-based studies has gained attention in Poland and Central-Eastern Europe^14^. This new focus is related mainly to the increasing awareness of the detrimental impact of ED and PE on health-related quality of life. Therefore, the aim of this study was to reliably evaluate the prevalence, effect on quality of life, and treatment-related behaviors for ED and PE in a population-representative sample of adult men aged ≥ 18 years in all geographical regions of Poland.

## Methods

This study was a population-based, representative, and cross-sectional investigation performed to provide estimates (stratified by age and place of residence) of ED and PE in Poland (running title: ED POLAND). We followed standardized guidelines and well-established recommendations for reporting observational studies^15^.

### Study design

We conducted computer-assisted web interviews (CAWI) between 1 September and 1 November 2022. Given the sensitive nature of the survey subject, and after considering the general applicability of large-scale studies on sex life, we implemented a CAWI system to guarantee the anonymity of the men respondents, to encourage their participation, and to avoid interviewer bias. We opted out of face-to-face interviews and telephone surveys because direct contact between interviewers and participants could have distorted our results. Moreover, previous studies have demonstrated that results from Internet surveys on sexual health (including the Men’s Attitudes to Life and Sexuality, the Global Study of Sexual Attitudes and Behaviors, and the Premature Ejaculation Prevalence and Attitudes) were not different from those obtained by other methods, such as telephone surveys or mailed questionnaires^4^. Because 93.33% of households in Poland had Internet access for 2022, with no significant differences between urban and rural areas (the Eurostat data and the Central Statistical Office of Poland data^16^), web interviews are justified for reliable population-based studies. Pilot web-surveys with cognitive debriefing comments (n = 200) were conducted before data collection to ensure that lay persons correctly understood the survey questions. All questions and terms were presented in Polish.

The most recent population census (2021) and the proportionate quota sampling method were employed to construct a population-representative sample of respondents (i.e., to ensure that the data would represent the general population)^17^. The quota controls included age and place of residence (for both geographical regions, all 16 states/voivodships in Poland, and type/size of places of living, urban and rural). For both urban and rural areas, we used definitions provided by the Central Statistical Office of Poland to appropriately cover these two types of regions: (1) urban areas (cities and towns) including areas located within the administrative boundaries of cities and towns; (2) rural areas (countryside) including areas outside the administrative boundaries of the cities, which consist of areas of rural gminas and rural parts of urban–rural gminas^18^.

The IPSOS Poland administered the survey^19^. The IPSOS is a research agency with relevant international quality certificates (PKJPA, PKJBI, OFBOR, ESOMAR) and prospective population-level databases of respondents, e.g., pre-existing Internet panels (at the time of this study, the total Polish IPSOS panel had about 50,000 members)^19^. The panel members, extracted by quota controls, were sent e-mail inviting them to participate in a confidential survey (5,800 members were extracted to receive the invitation). A unique Uniform Resource Locator (URL) was sent to each participant. All responses, during the entire study period, were collected on a web server with appropriate Secure Sockets Layer (SSL) certificate that authenticates a website identity and enables an encrypted connection. During data collection, there were regular quality-control and stratification checks. After data collection, post-stratification weights were calculated to correct any imbalances based on differences in response rates that were not detected during data collection. The weights were computed by ranking the completed interviews to the marginals for the matching variables (i.e., age and place of residence) before all statistical analyses. In addition, to exclude fraudulent responses, participants were excluded if they answered the survey too slowly (more than 10 min per page) and too quickly (a response time of less than 20% of the average response time).

### Measures

General demographic data were collected for each respondent, including age, level of education, employment status, and marital status.

ED was assessed using the five-item International Index of Erectile Function (IIEF-5)^20^, the most used^2^ and the consensual instrument for the evaluation of male sexual function^1^. PE was evaluated with the Premature Ejaculation Diagnostic Tool (PEDT)^21^, a valid screening instrument for PE^22^ that captures the essence of the multidimensional definition of PE^1^. Both questionnaires were rigorously translated and adapted into Polish^23,24^, and they are recommended by the European Association of Urology^1^.

Participants were also asked about their overall (‘If you were to spend the rest of your life in your current condition, how would you describe your overall well-being?’) and sex-specific (‘In the past 4 weeks, how were you satisfied with your sex life?’) quality of life, treatment-related behaviors (i.e., treatment seeking, receiving, satisfaction, and continuation), relevant comorbidities (i.e., arterial hypertension, myocardial infarction, any cardiac disease, diabetes, overweight, lipid disorders, stroke, any pulmonary disease, depression, any surgeries in abdomen or pelvis) and lifestyle habits (i.e., smoking, alcohol intake). All comorbidities were self-reported; we did not seek validation from medical records.

### Objectives

The primary study objective was to estimate the prevalence of ED and PE in men aged ≥ 18 years in Poland. Because previous studies were based on different thresholds of IIEF-5 and PEDT scores to assess ED and PE prevalence, we decided to adapt diverse definitions of various score intervals to make our results reliably comparable with multiple studies. For ED prevalence, definition I: IIEF-5 score of 21 points or less; definition II: IIEF-5 score of 16 points or less. For PE prevalence, definition I: PEDT score of 9 points or more; definition II: PEDT score of 11 points or more.

Secondary study objectives included the prevalence of different levels of ED severity (17–21 points from IIEF-5 indicating mild ED; 12–16 mild to moderate ED; 8–11 moderate ED; 5–7 severe ED), the effect of ED and PE on overall and sex-specific quality of life, the treatment behaviors for ED and PE, the correlations between ED/PE and relevant comorbidities/lifestyle habits, and the direct relation between ED and PE.

### Statistics

Quantitative variables were characterized by means, standard deviations, medians, minimum and maximum values (ranges), and 95% confidence intervals (CIs). Categorical variables were presented with numbers and percent. The Shapiro–Wilk test was used to test a normal distribution and the Leven (Brown-Forsythe) test was used to test the hypothesis of equal variances. Differences between two groups were tested with the Student t-test (in the absence of homogeneity of variance, the Welch test was used) or with the Mann–Whitney U test (model of unrelated variables) or with the Wilcoxon pair order test (model of related variables). Differences between more than two groups were tested with the F test (ANOVA) or Kruskal–Wallis test (model of unrelated variables) or with the Friedman test (model of related variables). When statistically significant differences between groups were obtained, post hoc tests were used (Tukey test for F test, Dunn test for Kruskal–Wallis test). For categorical variables, Chi-square test was applied (with Yates correction, assessment of Cochran conditions, and Fisher exact test, if applicable). The Pearson and/or Spearman correlation coefficients were calculated to establish relationships, strengths, and directions between variables. To analyse the effect of comorbidities and lifestyle habits on ED and PE, we also used logistic regression models. For regression models, we included results of the questionnaires as raw data (raw scores). Statistical significance was considered at *p* < 0.05. STATISTICA (StatSoft Inc., 2014, version 12.0.) was used to conduct data analysis.

For sample size calculation, we followed the methodology that was used in other population-based studies of ED/PE^25–28^. Thus, we computed the sample size based on the population age distribution, available from the recent census^17^, and expected ED/PE prevalence. Assuming a 95% confidence interval, we calculated that 2200 men in Poland would exceed the required sample size for estimating ED/PE prevalence. However, after analysing the recent census with general recommendations for future population-representative analyses, and after consulting with two independent teams of epidemiologists with relevant healthcare backgrounds, the sample size was raised to 3000 respondents. This increase in sample size provided smaller margins of error with no negative effects on any statistical analyses^17^. With a national sample of 3000, there was a 95% certainty that the overall survey results would be between ± 1–2% of what they would have been had we polled the entire adult male Polish population.

### Ethics approval

The study was performed in compliance with Good Clinical Practice and in accordance with the Declaration of Helsinki. The Research Ethics Committee of Jagiellonian University Medical College, Krakow, Poland approved the study (1072.6120.331.2021); in addition, the study was registered with ClinicalTrials.gov (NCT05462171).

### Consent to participate

All participants provided informed consent.

## Results

In all, 3001 men from across the country participated in the survey (response rate: 51.7%). Given the large sample size, the respondents were categorized into six age groups, 18–24, 25–34, 35–44, 45–54, 55–64, and ≥ 65 (10.3%, 19.5%, 23.4%, 18.3%, 19.3%, 9.2%, respectively, percent of all respondents). Most of the participants had at least secondary education, were employed, and married. More respondents lived in urban areas than in rural regions (75.8 vs. 24.2%). Table 1 presents detailed demographic characteristics.

**Table 1:** Basic demographics of the survey respondents.

	N (%)
Study participants	3001 (100%)
Age	
18–24	309 (10.3%)
25–34	586 (19.5%)
35–44	703 (23.4%)
45–54	549 (18.3%)
55–64	578 (19.3%)
65+	276 (9.2%)
Place of residence	
Urban (> 500,000 inhabitants)	424 (14.1%)
Urban (100,000–500,000 inhabitants)	629 (21.0%)
Urban (20,000–100,000 inhabitants)	742 (24.7%)
Urban (< 20,000 inhabitants)	479 (16.0%)
Rural	727 (24.2%)
Education	
Elementary	87 (2.9%)
Vocational	393 (13.1%)
Secondary	1343 (44.8%)
Higher	1178 (39.3%)
Employment	
Employed ^a^	2199 (73.3%)
Unemployed	292 (9.7%)
Pensioner/retired	444 (14.8%)
Other^b^	66 (2.2%)
Marital status	
Single	833 (27.8%)
Married or living with a partner	1964 (65.4%)
Separated or divorced	156 (5.2%)
Widower	48 (1.6%)

^a^Employed, self-employed, owners of own business/service/professional practice, or autonomous.

^b^Husband, stipendiary, and others (i.e., not in the above categories).

### Prevalence of ED

**Figure 1:**
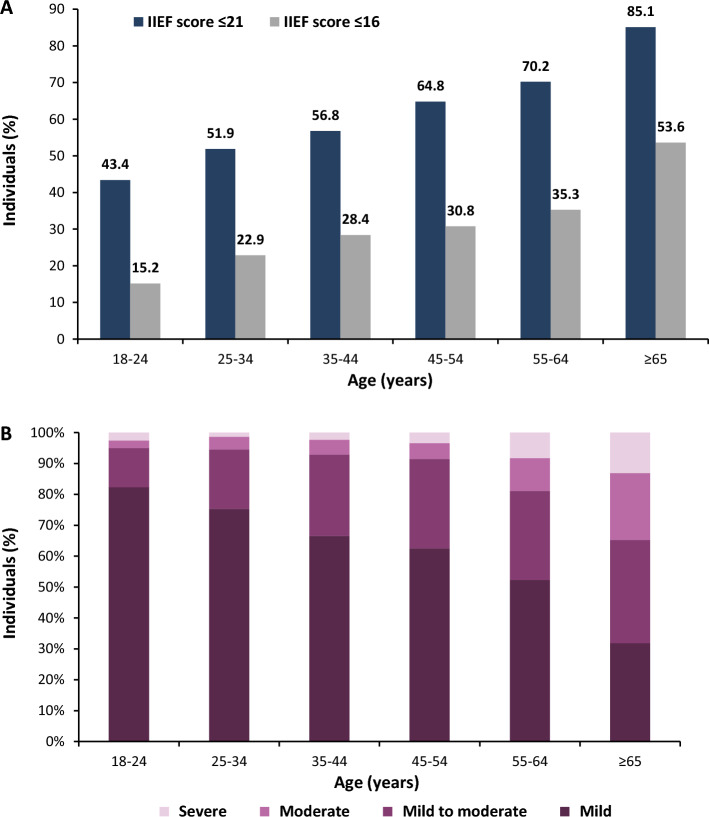
Prevalence (**A**) and distribution of severity (**B**) for ED based on the two study definitions: definition I—IIEF-5 score of 21 points or less; definition II—IIEF-5 score of 16 points or less. (N = 3001).

The prevalence of ED was 61.1% based on IIEF-5 score of 21 points or less (definition I), and the prevalence was 30.1% based on IIEF-5 score of 16 points or less (definition II). With both definitions, the prevalence of ED increased with age (Fig. 1; *p* < 0.0001, Spearman’s correlation coefficient R = −0.63). In all age groups, except for ≥ 65, ‘mild’ ED was the most prevalent ED severity category. In the ≥ 65 age group, ‘mild to moderate’ ED was the most prevalent group. In addition, the severity of ED increased with age (*p* < 0.0001, Spearman’s correlation coefficient R = −0.51).

**Figure 2:**
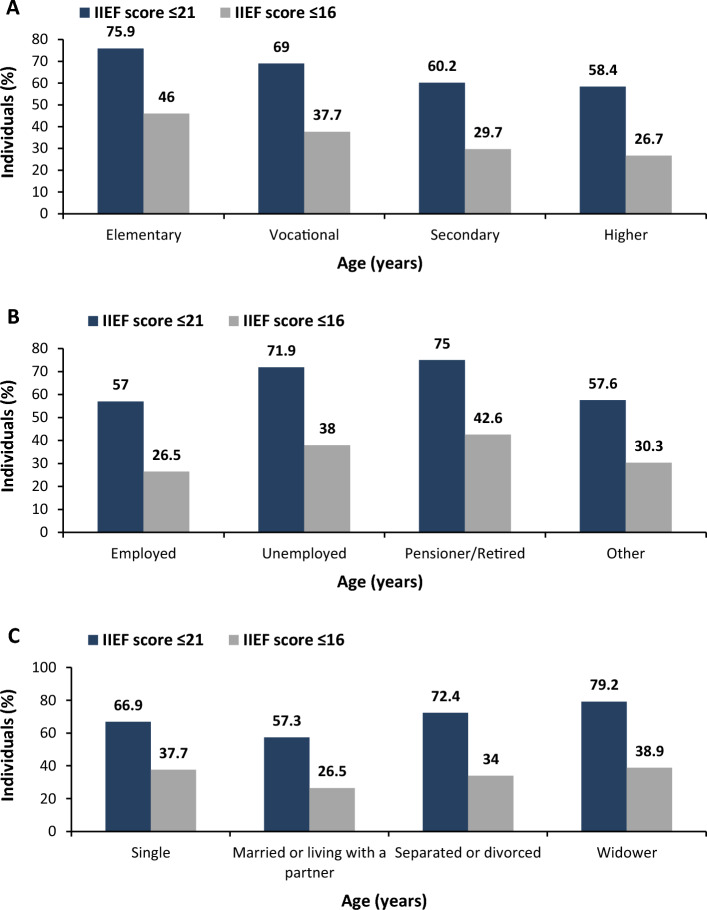
Education- (**A**), employment- (**B**), and marital status-specific (**C**) prevalence of ED based on the two study definitions: definition I—IIEF-5 score of 21 points or less; definition II—IIEF-5 score of 16 points or less.

We did not find any difference in ED prevalence across all geographical regions (voivodships) of Poland. There were also no significant correlations between ED prevalence and urban/rural status (definition I *p* = 0.4211, definition II *p* = 0.9101). However, other basic demographical parameters had important effects on ED prevalence. The prevalence of ED was lowest among men who had higher education, were employed, and married (Fig. 2; *p* < 0.0001 for all comparisons).

### Prevalence of PE

**Figure 3:**
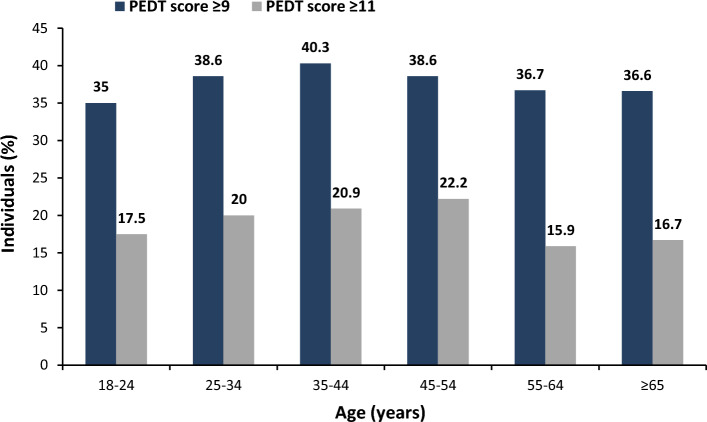
Prevalence of PE based on the two study definitions: definition I—PEDT score of 9 points or more; definition II—PEDT score of 11 points or more. (N = 3001).

The prevalence of PE was 38.1% based on PEDT score of 9 points or more (definition I), and the prevalence was 19.3% based on PEDT score of 11 points or more (definition II). There were no differences in PE prevalence between age groups (Fig. 3; definition I *p* = 0.6143, definition II *p* = 0.0623).

The PE prevalence did not differ across all geographical regions (voivodships) of Poland. There were also no significant correlations between PE prevalence and urban/rural status (definition I *p* = 0.6143, definition II *p* = 0.1673). We did not observe correlations between PE and education, employment, and marital status (*p* > 0.05 for all comparisons).

### Effect of ED and PE on overall quality of life

ED and PE had negative effects on overall quality of life. With definition I for ED, 48.7% of the respondents were ‘mixed’, ‘moderately dissatisfied’, or ‘dissatisfied’ with their overall quality of life. Corresponding data for ED definition II were 56.1%.

With definition I for PE, 53.9% of the respondents were ‘mixed’, ‘moderately dissatisfied’, or ‘dissatisfied’ with their overall quality of life. With definition II for PE, 58.7% of the survey participants responded similarly.

We also found that the more severe the ED or PE, the worse the overall quality of life (for ED: *p* < 0.01, Spearman's Rank correlation coefficient R = −0.33; for PE: *p* < 0.0001, Spearman's Rank correlation coefficient R = 0.30).

### Effect of ED and PE on sex-specific quality of life

The effects of ED and PE on sex-specific quality of life were more profound. According to definition I, 51.6% of the ED individuals reported that they were ‘mixed’, ‘moderately dissatisfied’, or ‘dissatisfied’ with the quality of their sex life. With ED definition II, 61.3% responded similarly.

Approximately 56% of the PE individuals classified by definition I reported that they were ‘mixed’, ‘moderately dissatisfied’, or ‘dissatisfied’ with the quality of their sex life. With PE definition II, 58.3% of the survey participants responded similarly.

We also found that the more severe the ED or PE, the worse the sex-specific quality of life (for ED: *p* < 0.01, Spearman's Rank correlation coefficient R = -0.37; for PE: *p* < 0.0001, Spearman's Rank correlation coefficient R = 0.28).

### Treatment behaviors for ED

Among respondents with IIEF-5 score of 21 points or less (definition I), 16.1% (n = 295) were seeking treatment and most of them (80.3%, n = 237) received treatment. For respondents with IIEF-5 score of 16 points or less (definition II), 23.4% (n = 211) were seeking treatment, and, again, many of these persons received treatment (78.2%, n = 165). For the respondents who received treatment, most continued treatment (87.3%, n = 207, for definition I; 89.1%, n = 147, for definition II) and most were satisfied with their therapy (80.2%, n = 190, for definition I; 77.0%, n = 127, for definition II).

We found that men with severe ED were more likely to seek treatment (*p* < 0.01), to continue the treatment (*p* = 0.0239), and to be satisfied with the treatment (*p* = 0.0037).

Urban/rural status did not affect any treatment behavior for ED (*p* = 0.6978).

### Treatment behaviors for PE

Among respondents with PEDT score of 9 points or more (definition I), 14.4% (n = 165) were seeking treatment and most of them (76.4%, n = 126) received treatment. For respondents with PEDT score of 11 points or more (definition II), 15.4% (n = 89) were seeking treatment, and, again, most of these persons received treatment (80.9%, n = 72). For the respondents who received treatment, most continued (84.9%, n = 107, for definition I; 77.8%, n = 56, for definition II) and were satisfied (77.8%, n = 98, for definition I; 75.0%, n = 54, for definition II).

We found that men with severe PE were more likely to seek treatment (*p* = 0.0001) and to continue the treatment (*p* = 0.0422).

There were no differences between urban and rural areas in treatment behaviors for PE (*p* = 0.7804).

### Correlations between ED/PE and relevant comorbidities/lifestyle habits

In a univariate logistic regression analysis, we found positive correlations between ED and arterial hypertension, myocardial infarction, any other cardiac disease, diabetes, overweight, lipid disorders, stroke, any pulmonary disease, depression, any surgeries in abdomen or pelvis, and alcohol intake. A multivariable logistic regression analysis disclosed correlations between ED and depression, any pulmonary disease, and lipid disorders. Detailed descriptions are presented in Table 2.

**Table 2:** Correlations between IIEF/PEDT scores and relevant comorbidities/lifestyle habits.

	IIEF score				PEDT score			
	Univariate logistic regression analysis		Multivariable logistic regression analysis		Univariate logistic regression analysis		Multivariable logistic regression analysis	
	OR (95%CI)	*P*-value	OR (95%CI)	*P*-value	OR (95%CI)	*P*-value	OR (95%CI)	*P*-value
Diabetes	2.63 (2.00;3.46)	< 0.0001			1.31 (0.78;2.20)	0.3053		
Depression	2.72 (2.06;3.59)	< 0.0001	1.62 (1.15;2.29)	0.0059	1.19 (0.72;1.96)	0.4915		
Any pulmonary disease	3.43 (2.42;4.86)	< 0.0001	1.70 (1.17;2.249)	0.0060	1.17 (0.69;1.98)	0.5575		
Any cardiac disease	2.92 (2.19;3.89)	< 0.0001			0.82 (0.48;1.40)	0.4688		
Arterial hypertension	1.83 (1.54;2.19)	< 0.0001			1.23 (0.75;2.01)	0.4060		
Lipid disorders	2.29 (1.85;2.83)	< 0.0001	1.29 (1.01;1.65)	0.0405	0.83 (0.50;1.39)	0.4841		
Myocardial infarction	3.73 (2.49;5.57)	< 0.0001			0.68 (0.36;1.29)	0.2376		
Stroke	4.20 (2.66;6.63)	< 0.0001			0.78 (0.43;1.42)	0.4161		
Smoking	1.14 (0.97;1.33)	0.1030			1.07 (0.66;1.73)	0.7982		
Overweight	1.33 (1.14;1.55)	0.0003			1.18 (0.72;1.91)	0.5145		
Alcohol intake	1.60 (1.30;1.97)	< 0.0001			1.02 (0.62;1.68)	0.9273		
Any surgery in abdomen or pelvis	1.74 (1.38;2.19)	< 0.0001			1.08 (0.65;1.79)	0.7770		

Cut-off value for alcohol intake was two or more drinks in a day.

In both univariate and multivariable logistic regression analyses, we did not observe any correlations between PE and relevant comorbidities/lifestyle habits (Table 2).

### Relationship between ED and PE

We found a significant correlation between ED and PE; the likelihood of PE increased with the severity of ED (Spearman's Rank correlation coefficient R = −0.25, *p* < 0.01). The results are presented in Table 3.

**Table 3:** Correlations between scores of IIEF and PEDT.

	PEDT > = 11	PEDT < 11	*P*-value	PEDT > = 9	PEDT < 9	*P*-value
IIEF			0.0001			0.0001
Severe ED	20 (3.5%)	76 (3.1%)		24 (2.1%)	72 (3.9%)	
Moderate ED	47 (8.1%)	153 (6.3%)		83 (7.3%)	117 (6.3%)	
Mild to moderate ED	155 (26.8%)	451 (18.6%)		348 (30.5%)	258 (13.9%)	
Mild ED	197 (34.1%)	735 (30.3%)		393 (34.4%)	539 (29.0%)	
No ED	159 (27.5%)	1008 (41.6%)		294 (25.7%)	873 (47.0%)	
IIEF < = 21			0.0001			0.0001
	419 (72.5%)	1415 (58.4%)		848 (74.3%)	986 (53.0%)	
IIEF < = 16			0.0001			0.0001
	222 (38.4%)	680 (28.1%)		455 (39.8%)	447 (24.0%)	

## Discussion

This study is the first population-representative epidemiological analysis of ED and PE conducted in a country of Central and Eastern Europe. We included respondents aged ≥ 18 years from all geographical regions of the country with adequate proportions of participants from urban and rural areas. We relied on instruments that are widely accepted and generally used for ED and PE evaluation, and we employed regular quality-control checks. Therefore, our investigation provides reliable, consistent, and valid population-level estimates of ED and PE prevalence, their effects on quality of life, and the treatment behaviors of affected individuals. We showed that ED and PE were highly prevalent and had negative effects on overall and sex-specific quality of life. However, most Polish men did not seek treatment for these conditions.

In a systematic review, Kessler et al. concluded that the global prevalence of ED was 13.1–71.2% as determined by studies based on the IIEF/IIEF-5^2^. Pooled results revealed the highest prevalence in Europe (up to 76.5%) and the lowest prevalence in South America (up to 55.2%). The authors suggested that the geographical disparities in ED prevalence were likely determined by genetic, environmental, lifestyle, and, importantly, cultural factors. Our observation (n = 3001) of ED in 30.1–61.1% of men aged ≥ 18 appears broadly comparable with data from population-based studies performed in other countries and regions. Similarly, our analysis confirmed a trend of increasing ED prevalence with increasing age. However, although ED prevalence varies across countries and regions, we need to recognize that ED affects people worldwide, and it is a serious public health concern. Moreover, we need to consider that, to some extent, all factors that influence ED (i.e., genetic, environmental, lifestyle, cultural) do not appear to affect ED occurrence. As the population has become older, and as obesity, diabetes, and hypertension become more prevalent, ED is becoming more common.

Until now, only one study was conducted to assess the population burden of ED in Poland. In 2006, Haczyski et al. analyzed the data reported by 340 randomly chosen physicians who were asked to interview five to twenty consecutive patients aged at least 40 years about the presence of ED^29^. From 3,552 returned questionnaires, the authors estimated an ED prevalence of 42.7%. However, since this finding was reported, the investigation has been questioned because the results were not based on a population-representative sample but were based on clinical convenience samples of special patient populations. Critically, results of studies of clinical populations cannot be extrapolated to the general population^25^. Some experts correctly suggest that men who frequently visit their physicians are more likely to be diagnosed with ED, often because of their comorbidities. Further, Haczynski et al. did not exploit a widely used and rigorously adapted instrument to assess ED; instead, they used their own questions that were not universally vetted. Although the study of Haczynski et al. laid the foundations for ED research in Poland, recent improvements in assessing erectile function have triggered the need to determine the true burden of ED in Poland. This determination requires the use of a reliable and generally accepted questionnaire for ED evaluation of a representative pool of men. Finally, there has never been an assessment of seeking professional help, treatment receiving, treatment satisfaction, and treatment continuation for ED in Poland. In view of all these concerns, we instituted the ED POLAND study to assess the true burden of ED in Poland.

The prevalence of PE has not been analysed to an extent similar to that of ED. Because there is lack of a clear and universally accepted definition of PE^30–32^, current diagnostic criteria are variable and largely rely on subjective measurements^3^. Further, experts recently proposed a new taxonomy for PE that encompasses other unaddressed and crucial clinical aspects of PE^33^. In addition, there is no consensus on normal ejaculatory latency time, perception of it varies by country, and diagnosis can further depend on whether it is assessed by patients or their partners. These limitations have impeded research on the prevalence of PE and the wide discrepancy in PE prevalence estimates has been well described^1^. The Global Study of Sexual Attitudes and Behaviors showed that approximately one-third of all men may have PE, but there were significant geographical differences^34^. The lowest prevalence was reported in the Middle East (12.4%), whereas the highest recorded prevalence occurred in Southeast Asia (30.5%). Notably, to assess PE, the study used only a single-item self-reported question about the reduction in ejaculatory latency time. Importantly, because bother is important for patient stratification, diagnosis, and treatment^1^, the current recommendation is that only individuals who report bothersome PE should be targeted (i.e., men with (1) reduced ejaculatory latency time, (2) negative personal consequences, such as distress, bother, frustration, and/or the avoidance of sexual intimacy, (3) and perceived lack of control over ejaculation)^1,33^. As a patient’s perception of PE is both an important component of diagnosis or treatment success and often subjective and highly individual, validated screening instruments that include relevant symptom burden of the disease seem to be optimal in population-based analyses^1^. Interestingly, the evolution in methodology of studies to assess PE prevalence has been noted; whereas some older studies estimated PE prevalence from single questions that asked about early ejaculation or that used crude time-intervals to ejaculation ^35,36^, recent studies were based on validated instruments for PE screening^37–39^. Therefore, we used the PEDT, a questionnaire that has demonstrated high sensitivity and specificity in identifying PE^1^, and its validity has been widely accepted in estimating PE prevalence in other large population-based analyses^1,37–39^. With the PEDT, we revealed a PE prevalence of 19.3–38.1%. In the United States Global Online Sexuality Survey study that also was based on the PEDT, 49.6% of respondents were classified as having PE^38^. In contrast, with the same instrument the PE prevalence was 12.1% in a Korean cohort of married couples^40^. These results agree with those reported from Italy and Australia, where PEDT-assessed PE prevalence was estimated to be 15.6%^41^ and 16%^42^, respectively. When we consider differences in methods of measuring PE and study populations, overall PE prevalence in our study appears consistent with international analyses. Moreover, in our study, we did not find an association between PE prevalence and age, an observation consistent with other global analyses^4,39,43^.

We confirmed that ED and PE had negative effects on overall and sex-specific quality of life. Importantly, the degree of bother caused by ED and PE may be independent of how researchers define ED and PE^44^. Although these conditions are not life-threatening, they result in withdrawal from sexual intimacy and may lead to anxiety, depression, anger, frustration, poor self-esteem, guilt, and lack of confidence. In additions, partners of ED and PE patients may be negatively impacted by ED and PE due to relationship difficulties and sexual dissatisfaction. The occurrence of ED may even negatively affect work productivity because men with ED have higher rates of absenteeism due to psychosocial and physical reasons^45^. Generally, many studies demonstrated that men with ED and PE experience a deterioration in psychological, social, physical, and economic well-being compared with men who do not have these conditions^44^.

The low rate of treatment seeking despite the negative effects of ED and PE on multiple aspects of life is a significant public health concern. We showed that only 16.1–23.4% of respondents with ED and 14.4–15.4% of those with PE were seeking treatment. Fortunately, most of these individuals received treatment, they continued the treatment, and they were satisfied with the treatment. The low percentage of people with ED and PE who sought treatment has been reported elsewhere. An Asian study showed that only 21% of men with ED sought medical care^46^. The results of European Burden of Illness Study (conducted in France, Germany, Italy, Spain, and the United Kingdom) showed that more than half of European men with self-reported ED did not discuss their condition with their physician^47^. For PE patients, a study from Turkey demonstrated that 22.4% of PE individuals were actively looking for treatment^48^. In Spain, a population-based study showed that only 16.7% of participants with PE were active in seeking information about the condition^49^. Barriers to seeking treatment include embarrassment, anxiety, social stigma, and treatment cost. In addition, often people are simply unaware that there may be treatment for their ailment; this issue is particularly important for men with PE. The knowledge about treatment-related behaviors may significantly support educational campaigns, health improvement programs, and resource allocation. Without adequate information about treatment, individuals cannot take optimal treatment seeking action. Finally, we can speculate that a true communication gap exists between Polish patients with PE or ED and their physicians.

We investigated a significant correlation between ED and PE at the population level. Therefore, our results support recent recommendations based on the new taxonomic clinical entity of the loss of control of erection and ejaculation^33^. Comorbidity of ED and PE has been reported^50,51^, and experts hypothesized a vicious cycle of ED and PE^33^. On the one hand, a man striving to control his ejaculation instinctively reduces his level of excitement, leading to ED. On the other hand, a man attempting to have an erection tries to increase his excitation, and the resulting overexcitement can undermine ejaculatory control^52^. Because the standard screening for ED and PE may lead to false positive results (up to 33%)^53^, experts propose introduction of the loss of control of erection and ejaculation as the primary definition of a disorder that is viewed as two deeply interrelated sexual symptoms^33^. Importantly, introducing this novel disorder may help to avoid consideration of ED and PE as completely separate entities.

A strong point of our study was an adequate representation of the entire population, with well-balanced demographic characteristics, and appropriate proportions of urban and rural area participants. We also employed the recent census to stratify our variables. Because our sample size was large (n = 3001), the results were within ± 1–2% of statistical error for the national population, making our analysis one of the most accurate in the current literature of ED and PE prevalence for a single country. We used well-established and rigorously adapted diagnostic tools for ED and PE evaluation together with many other questions. Before conducting the study, we extensively reviewed the literature to select appropriate instruments and to adapt appropriate cut-offs for these instruments. Although some investigators who performed population-level studies adapted the most widely used IIEF-5 threshold of 21 points or less to recognise ED^2^, other studies have been based on a lower threshold of 16 points or less, e.g., the Boston Area Community Health Survey^54^. The threshold of 16 points or less combines patients characterized as ‘no ED’ and ‘mild ED’ and makes a stricter estimate of ED, sometimes even considered as a clinically significant ED^25^. For the same reason, i.e., to make our results broadly comparable and to present population estimates that can be compared with multiple studies, we adapted two thresholds of PEDT: 9 or more (the threshold that combined respondents with ‘probable PE’ and ‘PE’^21^), and 11 or more (a stricter definition that comprised only respondents with ‘PE’^21^). Importantly, other investigators used this approach of two different PEDT cut-offs in population-based studies of PE^21,37,55,56^. Thus, our population-based analysis, that was planned in line with the current guidelines and recommendations, provides a clear view of the prevalence and related effects of ED and PE in Poland.

A main limitation of our study was the use of self-reports to measure ED and PE without medical evaluation. We did not also investigate the sexual orientation of respondents. In addition, we did not investigate the prevalence of lifelong and acquired PE. Nevertheless, because there is no consensus on definitions of lifelong and acquired PE, this assessment of lifelong and acquired PE was also omitted from other population-based studies^43^. To evaluate overall and sex-specific quality of life, we used only single questions. We did not ask about all factors that may interfere with the quality of life (e.g., psychological well-being, financial income, interpersonal relationships) as we had to limit the number of questions in our survey to foster respondent compliance. Because this study was conducted in Poland, results may not be generalizable elsewhere, especially for treatment-related behaviors. However, similar findings on behaviors related to treatment have been reported in other countries.

## Conclusions

This investigation was the first nationwide, population-representative epidemiological study of ED and PE performed in Poland. ED and PE were prevalent conditions and had negative effects on quality of life. However, less than one fourth of respondents with ED or PE were seeking treatment. Our findings agree with other epidemiologic reports of ED and PE conducted in different regions of the world, and our findings can support educational campaigns and health improvement programs for patients with ED and PE.

## Data Availability

All data generated or analysed during this study are included in this published article.
